# Texas and Mexico: Sharing a Legacy of Poverty and Neglected Tropical Diseases

**DOI:** 10.1371/journal.pntd.0001497

**Published:** 2012-03-27

**Authors:** Peter J. Hotez, Maria Elena Bottazzi, Eric Dumonteil, Jesus G. Valenzuela, Shaden Kamhawi, Jaime Ortega, Samuel Ponce de Leon Rosales, Miguel Betancourt Cravioto, Roberto Tapia-Conyer

**Affiliations:** 1 Sabin Vaccine Institute and Texas Children's Hospital Center for Vaccine Development, Section of Pediatric Tropical Medicine, Departments of Pediatrics and Molecular Virology & Microbiology, and National School of Tropical Medicine, Baylor College of Medicine, Houston, Texas, United States of America; 2 Laboratorio de Parasitología, Centro De Investigaciones Regional “Dr. Hideo Noguchi”, Autonomous University of Yucatan (UADY), Merida, Mexico; 3 Vector Molecular Biology Section, Laboratory of Malaria and Vector Research, National Institute of Allergy and Infectious Diseases, National Institutes of Health, Rockville, Maryland, United States of America; 4 Departamento de Biotecnología y Bioingeniería, Centro de Investigación y de Estudios Avanzados - Instituto Politécnico Nacional (CINVESTAV-IPN), Mexico City, Mexico; 5 Laboratorios de Biológicos y Reactivos de Mexico (BIRMEX), Mexico City, Mexico; 6 Instituto Carlos Slim de la Salud (ICSS), Mexico City, Mexico

*A consortium of institutions from Texas and Mexico has launched a new initiative for developing vaccines and other tools to control and eliminate neglected tropical diseases in Mesoamerica.*

The southern United States and northern Mexico not only share a border, they also share history, culture, and language. With its constant exchange of people and goods, the US–Mexico border region (of which Texas represents a large proportion) can be considered a single, unique, epidemiological unit with its own difficulties and challenges. Although Mexico and Texas have benefited from widespread economic development and with it improvements in life expectancy and overall public health, many diseases in a group of infections known as the neglected tropical diseases (NTDs) still remain highly endemic on both sides of the Texas–Mexico border. The NTDs are the most common infections of the poorest 120 million people in the Americas who live on less than US$2 per day [1]. They include ancient scourges such as hookworm and other soil-transmitted helminth infections, Chagas disease, amoebiasis, schistosomiasis, vivax malaria, leishmaniasis, and dengue [1]. Together, these NTDs produce a burden of disease in the western hemisphere that in certain regions even exceeds HIV/AIDS [1], while simultaneously trapping Latin America's “bottom 100 million” in poverty through their deleterious effects on child physical and intellectual development, pregnancy outcome, and worker productivity [2].

**Figure 1:**
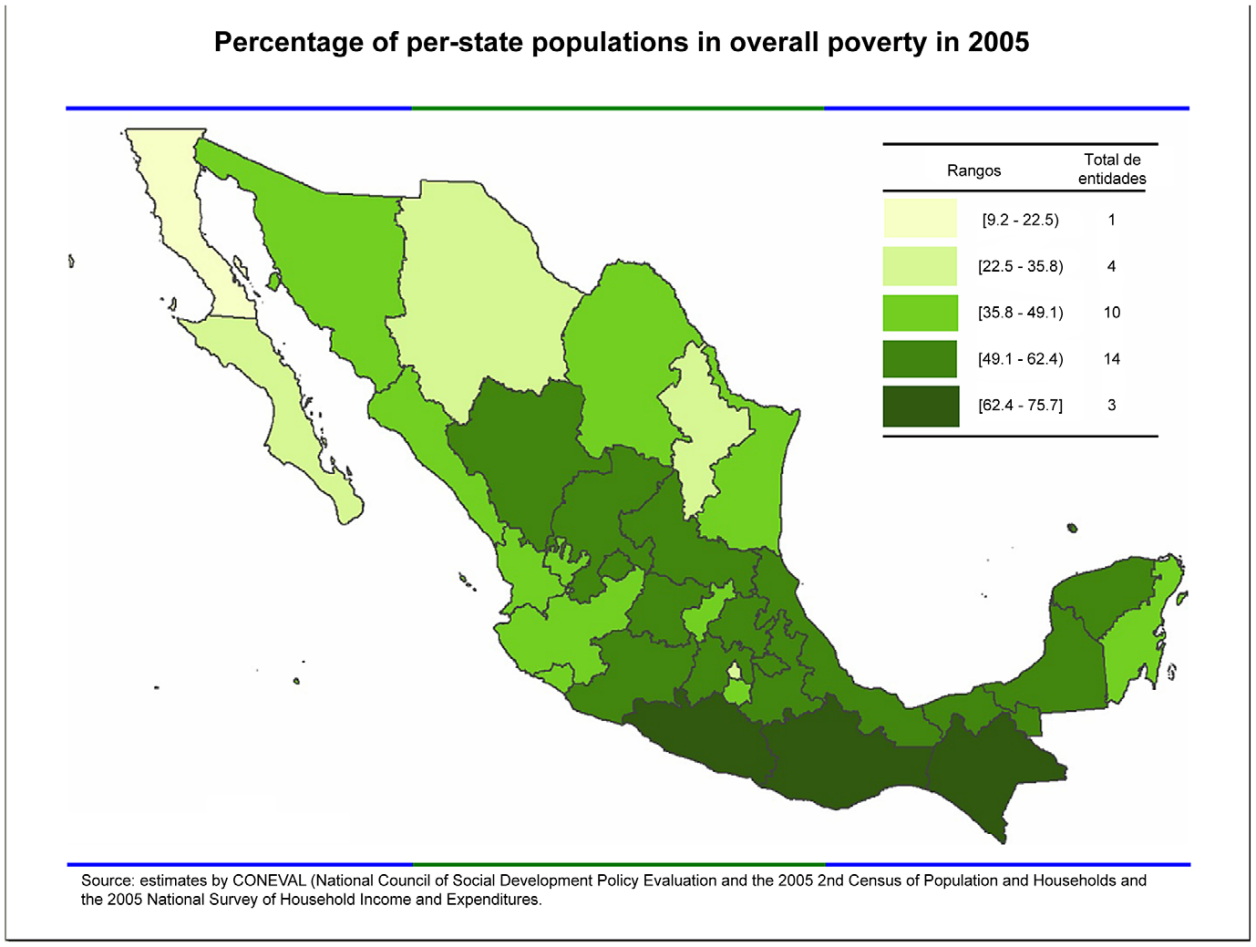
Poverty in Mexico by state. Map created by Usfirstgov with data from government website http://www.coneval.gob.mx/mapas/NACIONAL/Nacional.pdf, accessed at http://en.wikipedia.org/wiki/File:Overall_Poverty.PNG March 7, 2012.

With the exception of schistosomiasis and lymphatic filariasis, most of the major NTDs found in Latin America are also endemic to Mexico [3] (Table 1). Because poverty is an overwhelming risk factor for exposure to NTDs, the estimated 52 million people (46% of the population) who live on less than 2,114 pesos (about US$180) per month in urban areas or 1,329 pesos in rural areas and who lack at least one basic social right suffer the highest rates of these infections. The estimated 11 million people (10% of the population) who live in extreme poverty (less than 978 pesos in urban areas, less than 684 pesos in rural areas) and lacking at least one social right [3] are especially vulnerable [4]. Most of the NTDs occur in Mexico's poorest states, led by the contiguous southern states of Chiapas, Guerrero, and Oaxaca [4]. Overall, these three southern states, in addition to neighboring Campeche, Quintana Roo, Veracruz, and Yucatan, exhibit the lowest human development indices in Mexico [5] (Figure 1). Soil-transmitted helminth infections are among the most common NTDs in Mexico, led by trichuriasis (18 million cases), ascariasis (9 million cases), hookworm infection (1 million cases), and toxocariasis (number of cases not determined) [1], [6]. In rural Chiapas, *Necator americanus* hookworm infection is a significant cause of maternal-child anemia [7]. In addition, the incidence of cysticercosis, a soil-transmitted platyhelminth infection and a leading cause of epilepsy in Mexico, has been estimated at 0.4 per 100,000 people, with most of the cases in the southern states [5].

**Table 1:** The major NTDs of the Latin American and Caribbean region, Mexico, and Texas.

Disease	Estimated Number of Cases in Latin America and the Caribbean [1], [5]	Estimated Number of Cases in Mexico [3], [5], [17], [18]	Disease Endemic to Texas?
Trichuriasis	100 million	18 million	Unknown
Ascariasis	84 million	9 million	Unknown
Hookworm	50 million	1 million	Previously endemic
Amoebiasis	Not determined	8–9 million	Unknown
Chagas disease	8–9 million	2–6 million	Yes – up to 267,000 cases
Schistosomiasis	2–7 million	None	None
Blinding trachoma	1.1 million	<1,000	None
Vivax malaria	<0.9 million reported cases in 2004	<3,000 cases reported in 2005 and 2009; <1,000 cases up to week 44 in 2011^a^	None
Lymphatic filariasis	0.7 million	None	None
Dengue	0.5 million	27.2 cases per 100,000	Yes
Cysticercosis	0.4 million	<10,000 reported; incidence of 0.4 per 100,000	Yes
Leishmaniasis	67,000	<10,000 reported	Yes
Leprosy	33,953 registered cases	478 registered cases at the end of the first quarter of 2011	Unknown
Brucellosis	Not determined	24,000 reported; incidence of 2–3 per 100,000	Unknown
Leptospirosis	Not determined	Not determined	Unknown
Onchocerciasis	Near elimination	Near elimination	None

a The number of cases of malaria in 2005 is published in [5]. These numbers were updated in 2009 in an unpublished report (Secretaria de Salud, Anuario de Morbilidad 2009, Mexico D.F., 2010) and up to week 44 in 2011 (Secretaría de Salud, Boletin Epidemiologia, Semana 44, Mexico D.F., 2011).

Equally important to Mexico are the major vector-borne NTDs, led by up to six million or more cases of Chagas disease (American trypanosomiasis caused by *Trypanosoma cruzi*), which are found primarily in the states of Chiapas, Oaxaca, Puebla, Veracruz, and Yucatan [8]. In addition to transmission by triatomine kissing bugs, both congenital infections and transfusion-related *T. cruzi* infections also occur [9]. Leishmaniasis is also endemic. Cutaneous leishmaniasis (CL) is responsible for 99% of the cases, mostly caused by *Leishmania mexicana* [5]. CL is hyperendemic in the state of Tabasco in association with the cocoa industry there. There are five possible vector species of the genus *Lutzomyia* responsible for the transmission of CL; *Lutzomyia olmeca* is the predominant species on the Yucatan peninsula and the incriminated vector in the state of Tabasco for CL [10]. Visceral leishmaniasis has also been reported annually from Chiapas for almost the last 20 years [5]. Unfortunately, most leishmaniasis cases are still underreported in Mexico. Vivax malaria is found predominantly in the poorest states of Chiapas and Oaxaca, as well as in Sinaloa, Chihuahua, Durango, and Tabasco, although only a small percentage of these cases are reported [3], [5]. Fewer than 3,000 cases of vivax malaria were reported in Mexico in 2005 and 2009 (and less than 1,000 cases in 2011) [5]. Onchocerciasis is traditionally endemic in three distinct foci in Mexico, i.e., Oaxaca, northern Chiapas, and southern Chiapas [11], [12], although in 2010 it was reported that no transmission has been detected in the first two foci [11], [12]. Dengue remains highly endemic in Mexico [13], with dengue virus type 2 (DENV-2) representing the predominant serotype [5]. However, all four serotypes of dengue are now present in Mexico, due to reintroduction of DENV-1 and DENV-4 from Central America, and rates of severe dengue have increased significantly since 2000 [5]. West Nile virus infection has also been reported in Mexico [5].

Among the protozoan NTDs, amoebiasis and giardiasis are each widespread enteric infections [14], [15], and toxoplasmosis is an important protozoan infection and a risk for pregnant women [16], although national prevalence data are not available for any of these conditions. Brucellosis, leprosy, leptospirosis, and trachoma are the major bacterial NTDs [3]. The incidence of human brucellosis is two to three cases per 100,000 people, with the largest number in the states of Coahuila, Nuevo Leon, Sinaloa, and Zacatecas, mostly from contaminated milk and milk products [5]. Mexico is one of three Latin American countries (the others being Brazil and Guatemala) with endemic trachoma [17]. The disease is endemic in five municipalities of Los Altos-Chiapas, with a control program in place to search for cases house by house [5]. The World Health Organization reported 478 cases of registered leprosy in Mexico at the end of the first quarter of 2011 [18]. Canine rabies is still reported in Mexico, with two deaths from dog bites between 2000 and 2005 [5]. Overall, there is a need to increase our understanding of the epidemiology for NTDs in Mexico.

**Figure 2:**
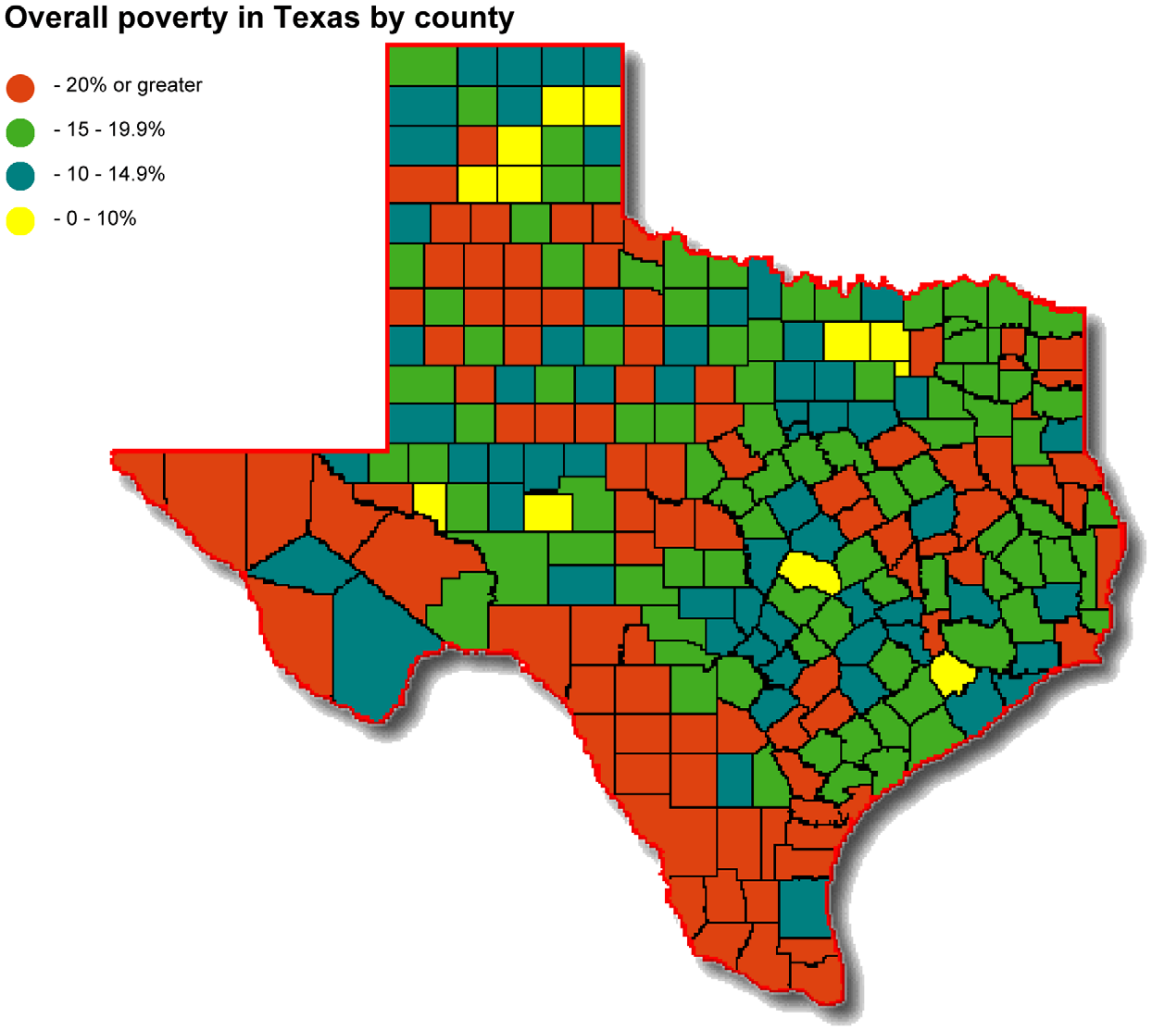
Figure created at diy.net with data from US Census Bureau 2010 Small Area Income and Poverty Estimates accessed at http://www.census.gov/did/www/saipe/county.html March 6, 2012.

Across the border, the state of Texas is neither immune to poverty nor to the NTDs. Indeed, at a 17% poverty rate, Texas has a significantly higher rate than the overall 14% poverty rate in the United States [19]. With 4.15 million people living below the poverty line, Texas may have the largest number of poor people of any state in the US [19]. The poverty rates are highest among Hispanic (26%) and African-American minorities (23%) and among children under the age of five (all races) (28%) [19]. Poverty in Texas is concentrated in South Texas, especially along the border with Mexico (Figure 2).

Emerging evidence over the last few years has revealed a hidden burden of NTDs and related neglected infections of poverty in Texas [20]. Among the helminthic NTDs during the first half of the 20th century, hookworm infection was hyperendemic in east Texas [21]–[23], with prevalence rates as high as 84% in the Piney Woods counties of Sabine, San Augustine, Jasper, and Newton [21]. The infection was found in association with sandy soils present near rivers [21]. Hymenolepiasis was also determined to be widespread during this period [21]. However, no studies of hookworm in Texas have been reported in the last 50 years, and it is not known whether hookworm or other intestinal helminth infections such as ascariasis and trichuriasis remain endemic in the poorest rural areas of east Texas. In contrast, it is now well established that cysticercosis is a leading cause of epilepsy among Hispanics living in Texas [24], [25]. Up to 169,000 cases of cysticercosis are estimated to occur in the US, with Texas and California most likely representing the greatest share of the disease burden from this condition [20]. Toxocariasis, a zoonotic larval helminth infection, is widespread among African-American and Hispanic populations in the American South [20]; based on its prevalence among urban dogs in Houston and presumably elsewhere [26], it is likely that toxocariasis is also widespread in Texas. This condition has been linked to asthma and developmental delays [27].

Several important vector-borne NTDs have recently emerged in Texas, led by Chagas disease, leishmaniasis, and dengue [20]. Up to 267,000 cases of Chagas disease are believed to occur in Texas [28], although this figure may be an overestimate [29]. The largest number of US cases of Chagas disease may occur in Texas as a result of human migrations from Mexico in addition to autochthonous transmission [30], [31]. Infected vectors or hosts are present in 82 of the 254 counties of Texas [30]; they include wild zoonotic reservoirs such as armadillos, coyotes, raccoons, opossums, and rodents of the genus *Neotoma*, canine reservoirs, and up to 11 species of kissing bugs, including three major *Triatoma* species, i.e., *Triatoma gerstaeckeri*, *Triatoma sanguisuga*, and *Triatoma leticulara* [30], [31]. Two major genotypes of *T. cruzi*, i.e., TcI and TcIV, have been reported from the US [31]. Although only four cases of autochthonous infections have been recorded in Texas [31], this number is likely to be an underestimate of the true number of cases [32], with the additional possibility of congenital infections [9], [31], [33]. A risk analysis based on the ecology and incidence of *T. cruzi* infection among vectors and animal reservoirs indicates that the greatest risk of Chagas disease occurs in the south Texas counties of Cameron, Nueces, Kleberg, Hidalgo, Jim Wells, Willacy, Medina, Dimmit, Frio, and Bandera, with expectations of *T. cruzi* exposures and infections among the major population centers in Dallas, Houston, and San Antonio [30]. CL from *L. mexicana* infection (which is transmitted by *Lutzomyia* sand flies) is endemic in south-central Texas [34], [35], with at least nine autochthonous cases reported from north Texas [36]. The suspected (but unproven) vector for CL in this area is *Lutzomyia diabolica*, but more entomological and parasitological studies are required to define the sand fly vector species responsible for the transmission of CL. Dengue is also endemic along the border with Mexico, with an estimated 2% seroprevalence in Brownsville [37]. The major risk factors along the Texas–Mexico border include low weekly family income, absence of air conditioning and window screens, and inadequate sanitation [37], [38]. Such conditions are found more commonly across the border from Brownsville in Matamoros, Tamaulipas State, Mexico, where the seroprevalence is almost four times higher [37]. Of particular concern is the observation that a more virulent and transmissible genotype of dengue serotype 2 has been introduced into the Texas–Mexico border area [39]. Approximately 100,000–200,000 cases of dengue have been estimated to occur among the Mexican-American population in the US [20].

The high prevalence and incidence of the major NTDs in both Mexico and south Texas afford an opportunity for joint cooperation to address the highest prevalence conditions, especially Chagas disease, CL, dengue, and the soil-transmitted helminth infections. For each of these NTDs there are widely disparate disease estimates available, and this situation suggests some urgency for programs of active surveillance based on seroprevalence and other diagnostics studies. There is an equally urgent need to determine the major mechanisms of transmission, which for Chagas disease would also include the transmission from dogs and other canines, estimates of the extent of congenital infection, and the incidence of infection acquired through blood transfusion. Such efforts should include studies to screen for congenital Chagas disease transmission in hospitals with a high proportion of women from Latin America [31]. Among the recommendations recently suggested for the control of Chagas disease in Texas is the need to make Chagas disease reportable (as it has been in Arizona and Massachusetts [30]), to carry out serological studies of human and canine populations, to monitor the extent of *T. cruzi* infection in rodents and other wild zoonotic reservoirs, and to undertake widespread testing of blood donors and other at-risk populations [30]. Similar programs of surveillance and transmission dynamics are also required for CL, dengue, and helminth infections [20]. Given the risks of Chagas disease (including congenital Chagas disease) in Mexico and the US, there is an urgent need to educate cardiologists, obstetricians, and other health care providers about the likelihood of this and other neglected infections of poverty [31].

There is also an urgent need to develop alternative control tools for the major NTDs. Recently, the Instituto Carlos Slim de la Salud (Carlos Slim Health Institute) launched a joint US–Mexico initiative to develop NTD vaccines, beginning with Chagas disease and CL [2], [40]. The Iniciativa Slim para el desarollo de vacunas contra enfermedades tropicales (Slim Initiative for developing tropical disease vaccines) is focusing its initial efforts on developing a therapeutic vaccine for Chagas disease with an emphasis on two recombinant *T. cruzi* antigens, Tc24 and TSA-1 [2], [41], and a preventative CL vaccine against *L. mexicana* infection comprised of a recombinant *L. mexicana* nucleoside hydrolase [42]–[44] and a recombinant *Lutzomyia* sand fly salivary antigen [45]–[47]. Such antigens would be developed jointly by institutions based in Texas and Mexico, in addition to the US National Institutes of Health, with manufacture under cGMPs (current good manufacturing practices) by Laboratorios de Biológicos y Reactivos de Mexico (Birmex) [48], the major biologics manufacturing organization of the Mexican government.

In parallel, a newly established Section of Pediatric Tropical Medicine at Texas Children's Hospital of Baylor College of Medicine has linked with other institutions within the Texas Medical Center and the Sabin Vaccine Institute to develop vaccines and other appropriate technologies for NTDs [49], [50]. These organizations provide the basis for a new National School of Tropical Medicine recently established at Baylor College of Medicine [51], [52]. Under the auspices of the Slim Initiative, it is anticipated that these joint activities might lead to a new generation of NTD vaccines for hookworm, Chagas disease, and leishmaniasis. Such technologies are sometimes referred to as “antipoverty vaccines” for their potential impact to not only improve health, but also to help lift Latin America's bottom 100 million out of poverty [53], [54]. Ultimately, an all-out assault on the NTDs is necessary if we hope to achieve elimination of these ancient conditions in the coming decade.
